# Supplementary material for: Differential trafficking of ligands trogocytosed via CD28 versus CTLA4 promotes collective cellular control of co-stimulation
Source: Nat Commun. 2022 Oct 29;13:6459. doi: 10.1038/s41467-022-34156-1 (PMC9617924; doi:10.1038/s41467-022-34156-1)
Supplement: Supplementary file 1 — Supplementary Information [file 41467_2022_34156_MOESM1_ESM.pdf]

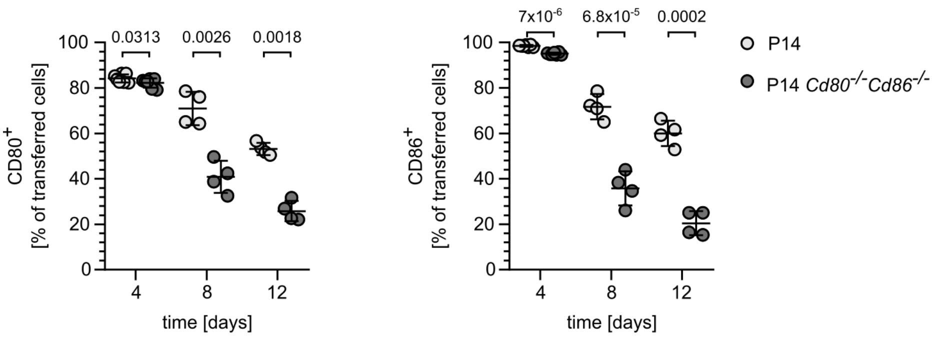

Supplementary Figure 1: Related to Figure 1
Expression of CD80 and CD86 on adoptively transferred WT and *Cd80*$^{-/-}$*Cd86*$^{-/-}$ P14 T cells upon LCMV-infection. Plots depict proportion of CD80+ (left) or CD86+ (right) cells in spleen.
Statistics: Pooled data from 2 (day 4) – 1 (day 8, 12) independent experiments. Two-sided Wilcoxon test: day 4 CD80+ cells: p-value 0.0313; Two-sided paired t test: day 8 CD80+ cells: p-value 0.0026; day 12 CD80+ cells: p-value 0.0018; day 4 CD86+ cells: p-value: 7x10-6; CD8 CD86+ cells: p-value: 7x10-5; day 12 CD86+ cells: p-value 0.0002. (day 4: n=7 animals, days 8 and 12: 4 animals). Source data are provided as a Source Data file.

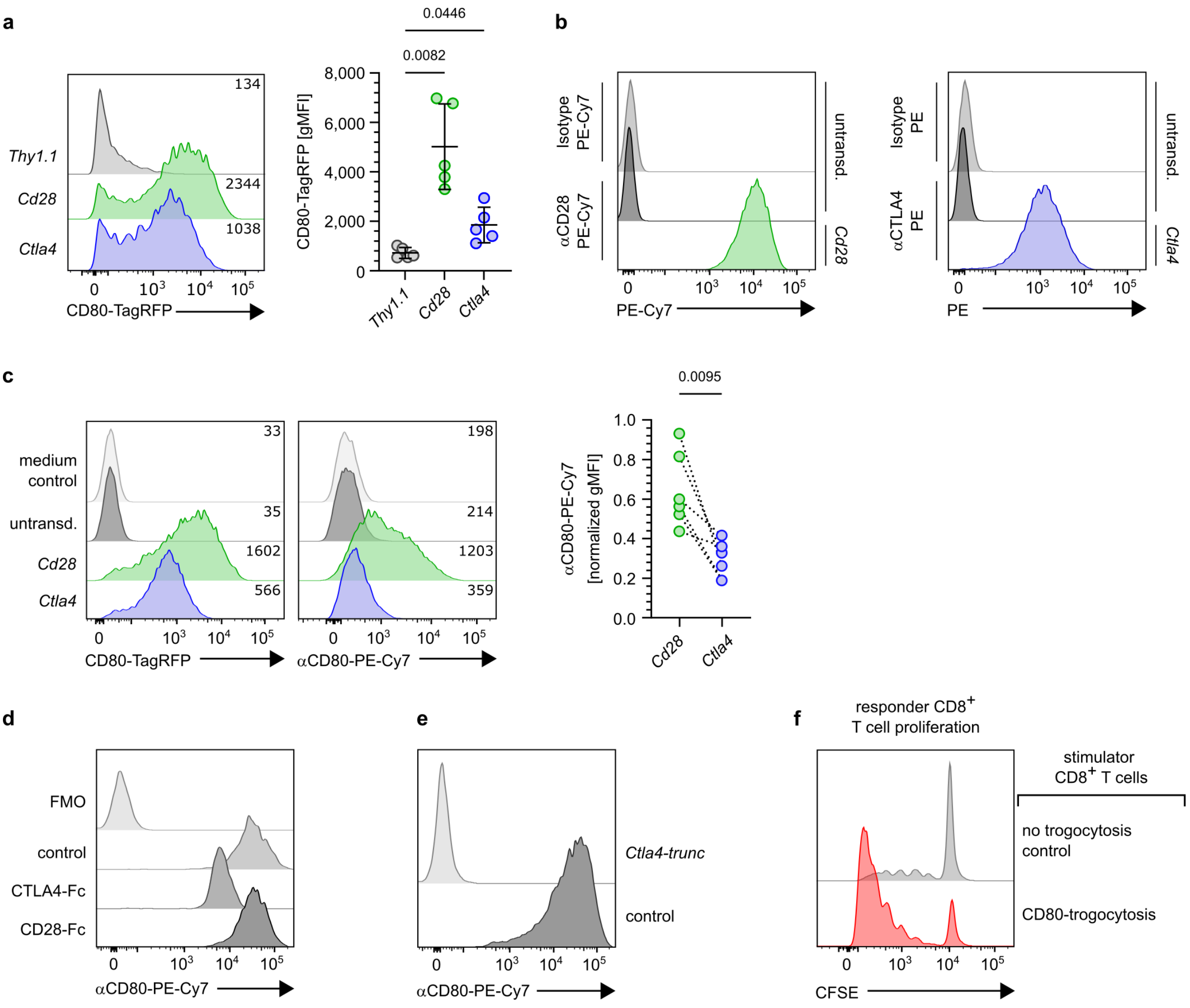

Supplementary Figure 2: Related to Figure 2

(a) Transduction with CD28 or CTLA4 suffices to enable B lymphocyte trogocytosis of CD80. Representative histograms (left) and quantification of CD80-TagRFP gMFI (right) acquired by *Thy1.1*- (control), *Cd28*- or *Ctla4*-transduced *Cd80*$^{-/-}$*Cd86*$^{-/-}$ B220^{+} B cells upon 2h co-culture with MEF cells expressing CD80-TagRFP. Data pooled from 5 independent experiments. (b, c) T cells lacking CD28 or CTLA4 are unable to trogocytose CD80. (b) Expression of CD28 and CTLA4 by untransduced, *Cd28*- or *Ctla4*-transduced CD8^{+} 58αβ A2 T cells. (c) Trogocytosis and αCD80 surface stain of CD80-TagRFP acquired by untransduced, *Cd28*- or *Ctla4*-transduced CD8^{+} 58αβ A2 T cells after 6h co-culture with CD80-TagRFP expressing CHO cells or cultured in the absence of CHO cells (medium control). Representative histograms (left, middle) and quantification of pooled data (right) from 6 independent experiments. To correct for different trogocytosis rates between CD28 and CTLA4, surface CD80 levels were normalized to total amount of acquired CD80-TagRFP (CD80-PE-Cy7 gMFI / TagRFP gMFI). (d) Competition between CD80 antibody staining and CTLA4-Fc. Representative histograms of Anti-CD80 surface staining of CD80 expressing MEF cells pre-incubated with PBS (control), CD28-Fc or CTLA4-Fc. (e) CD80-CTLA4 *cis*-interaction impairs Anti-CD80 antibody binding. MEFs expressing CD80 were either mock-transduced (control) or transduced with a truncated, cell surface-targeted CTLA4-variant (lacking the IC domain), followed by staining with an Anti-CD80 PE-Cy7 antibody. Note that co-expression of the cell-surface targeted CTLA4 variants abrogates antibody-based CD80 staining, indicating that CD80 and CTLA4 can interact in *cis*. (f) T cell trogocytosis of CD80 enables co-stimulation of neighbouring cells. Antigen-stimulated Cd80$^{-/-}$Cd86$^{-/-}$ T cells were co-cultured with CD80-mScarlet expressing MEFs, FACS-sorted for mScarlet-expression and co-cultured with CFSE-labeled Cd80$^{-/-}$Cd86$^{-/-}$ P14 CD8+ T cells in presence of gp33-antigen. Plots depict day 3 T cell CFSE dilution profiles. Plot depicts representative data of 2 independent experiments

Statistics: S2a: Pooled data of 5 independent experiments. Repeated measures one-way ANOVA with Dunnett's T3 correction. (n=5 biologically independent samples per group). S2b,c: Histograms: representative data from 1 of 6 independent experiments. Plot: pooled data from 6 independent experiments. Two-sided paired t test. (n=6

biologically independent samples per group). S2d,e,f: representative data from 1 of 2 independent experiments. (n=1 biological independent sample per group). Source data are provided as a Source Data file.

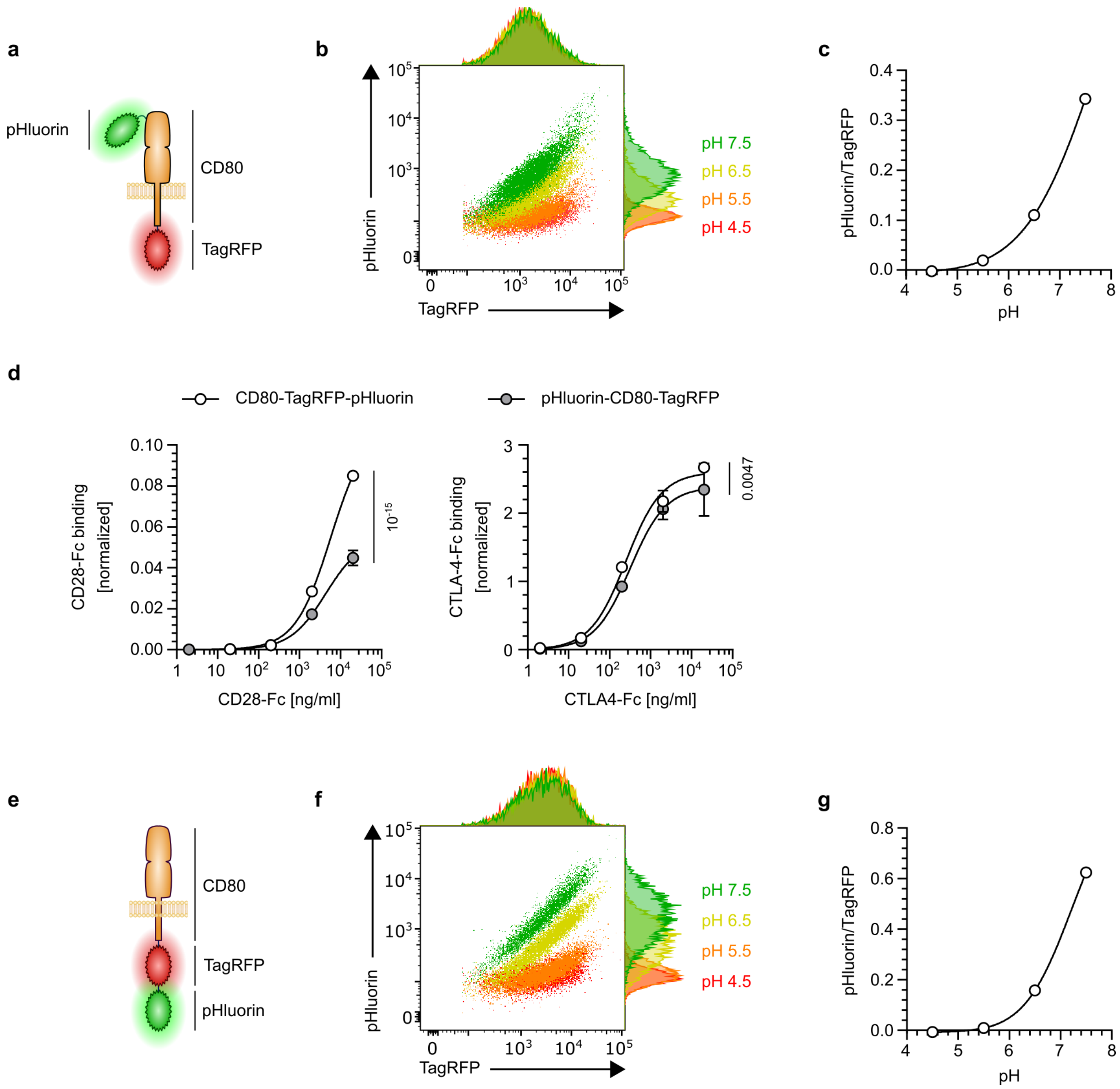

Supplementary Figure 3: Related to Figure 3 and 4
(a-c) Ratiometric reporter enables determination of pH experienced by the extracellular domain of CD80 upon trogocytosis. (a) Reporter setup consisting of CD80-TagRFP fusion protein with extracellular pH-sensitive pHluorin-tag. (b) Flow cytometric analysis of trogocytosed ratiometric pH-reporter at different pH-levels. CD28 expressing 58αβ A2 T cells were co-cultured with CHO cells expressing pHluorin-CD80-TagRFP and subsequently exposed to cell permeable pH calibration buffers ranging from pH 4.5 to 7.5. (c) Standard curve obtained by plotting pHluorin gMFI / TagRFP gMFI ratios against pH-levels of calibration buffers enables extrapolation of the average pH experienced by CD80 EC domain upon trogocytosis. Data representative of 3 independent experiments. (d) Extracellular pHluorin-tag reduces but does not abrogate binding of CD80 to CD28 and CTLA4. CHO cells expressing pHluorin-CD80-TagRFP or CD80-TagRFP-pHluorin (control) were incubated with CD28-Fc (left) or CTLA4-Fc (right), followed by Anti-IgG-AF647 antibody staining and flow cytometric analysis. Plots depict antibody-derived fluorescent signal normalized to transgene expression (TagRFP gMFI). Error bars depict mean ± SD of technical replicates. (e, f) Modified ratiometric reporter enables determination of pH experienced by the intracellular domain of CD80 upon trogocytosis. (e) Reporter setup consisting of CD80-TagRFP fusion protein with intracellular pH-sensitive pHluorin-tag. (f) Flow cytometric analysis of trogocytosed ratiometric pH-reporter at different pH-levels. CD28 expressing 58αβ A2 T cells were co-cultured with CHO cells expressing CD80-TagRFP-pHluorin and subsequently exposed to cell permeable pH calibration buffers ranging from pH 4.5 to 7.5. (g) Standard curve obtained by plotting pHluorin gMFI / TagRFP gMFI ratios against pH-levels of calibration buffers enables extrapolation of the average pH experienced by CD80 IC domain upon trogocytosis. Data representative of 3 independent experiments
Statistics: S3c: representative data from 1 of 3 independent experiments. Curve fit: sigmoidal Sigmoidal, 4PL. (n=1 biologically independent sample per group). S3d: data from 1 experiment. Extra sum-of-squares F test. p

value: CD28-Fc binding: 1x 10^{-15}, CTLA-4-Fc binding: 0.0047. (n=3 biologically independent samples per group). S3g: representative data from 1 of 3 independent experiments. Curve fit: sigmoidal Sigmoidal, 4PL. (n=1 biologically independent sample per group.). Source data are provided as a Source Data file.

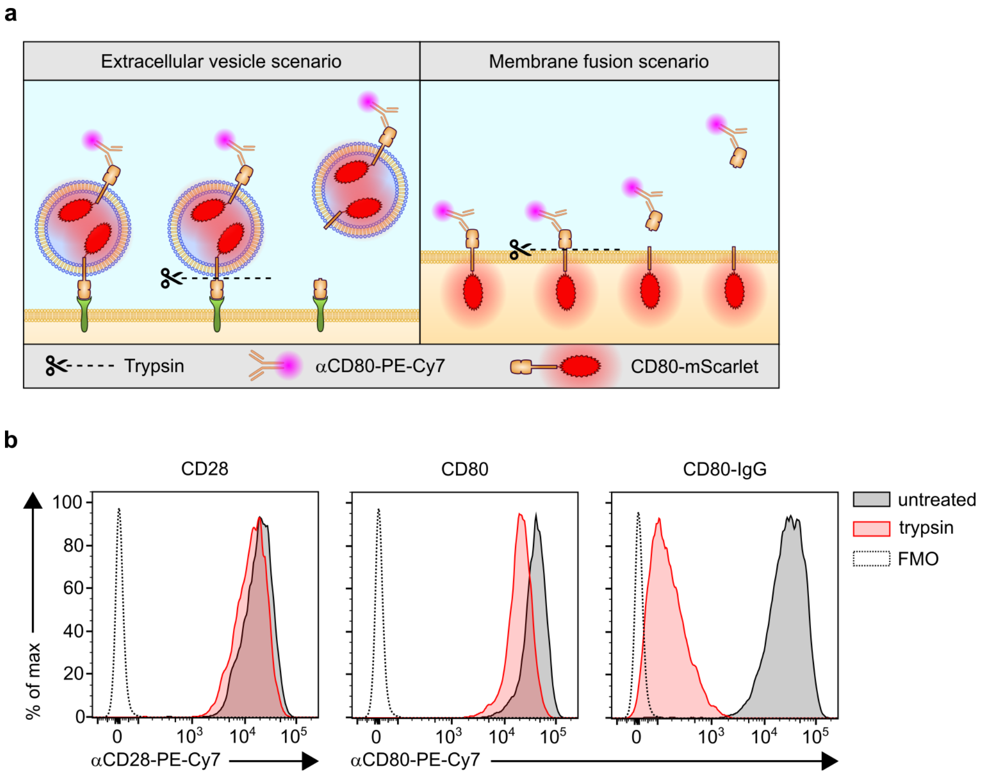

Supplementary Figure 4: Related to Figure 4
(a) Experimental approach to distinguish whether trogocytosed CD80 locates to membranous vesicles at the surface of (and within) recipient cells (extracellular vesicle scenario, left) or becomes integrated into recipient cell membrane (= membrane fusion scenario, right). The assay is based on comparing the effects of proteolytic cleavage of the CD80 extracellular domain on the signal emitted by a fluorescent protein attached to the CD80 intracellular domain. In a "membrane fusion scenario" this fluorescent signal should be unaffected by the proteolytic cleavage, whereas in an "extracellular vesicle scenario" it should be reduced. Loss of binding of fluorescent antibodies to the CD80 extracellular domain serves as a control for efficient proteolytic cleavage of CD80. (b) Determination of protease sensitivity of CD28, CD80 and CD80-IgG. CHO cells expressing CD28, CD80 or a CD80 variant with an IgG-constant domain inserted into the CD80 stalk region (CD80-IgG) were treated with trypsin. Subsequently cells were stained with fluorescent antibodies against CD28 or CD80 and analyzed by flow cytometry. Note that while CD28 and CD80 are largely resistant to trypsin, CD80-IgG is sensitive to cleavage by this protease.

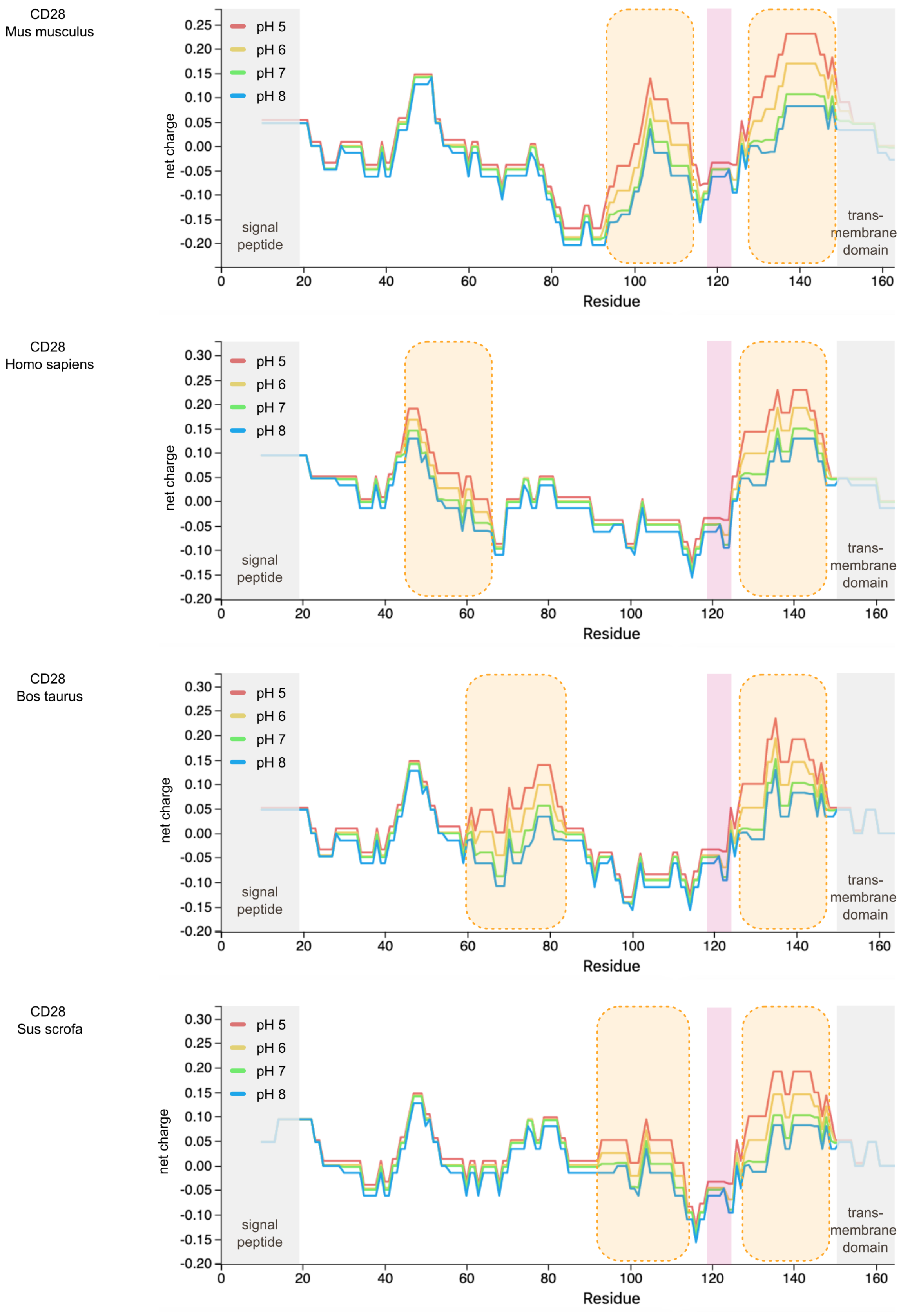

Supplementary Figure 5: Related to Figure 6
Identification of a conserved acid-sensitive region in the CD28 extracellular domain.
Charge profiles of the CD28 extracellular domain of different species from pH 5 (red) to pH 8 (blue) in steps of pH 1. Sequence regions that exhibit the most pronounced change in charge are marked by orange rectangles. The MYPPPY-ligand binding motif is tinted pink.

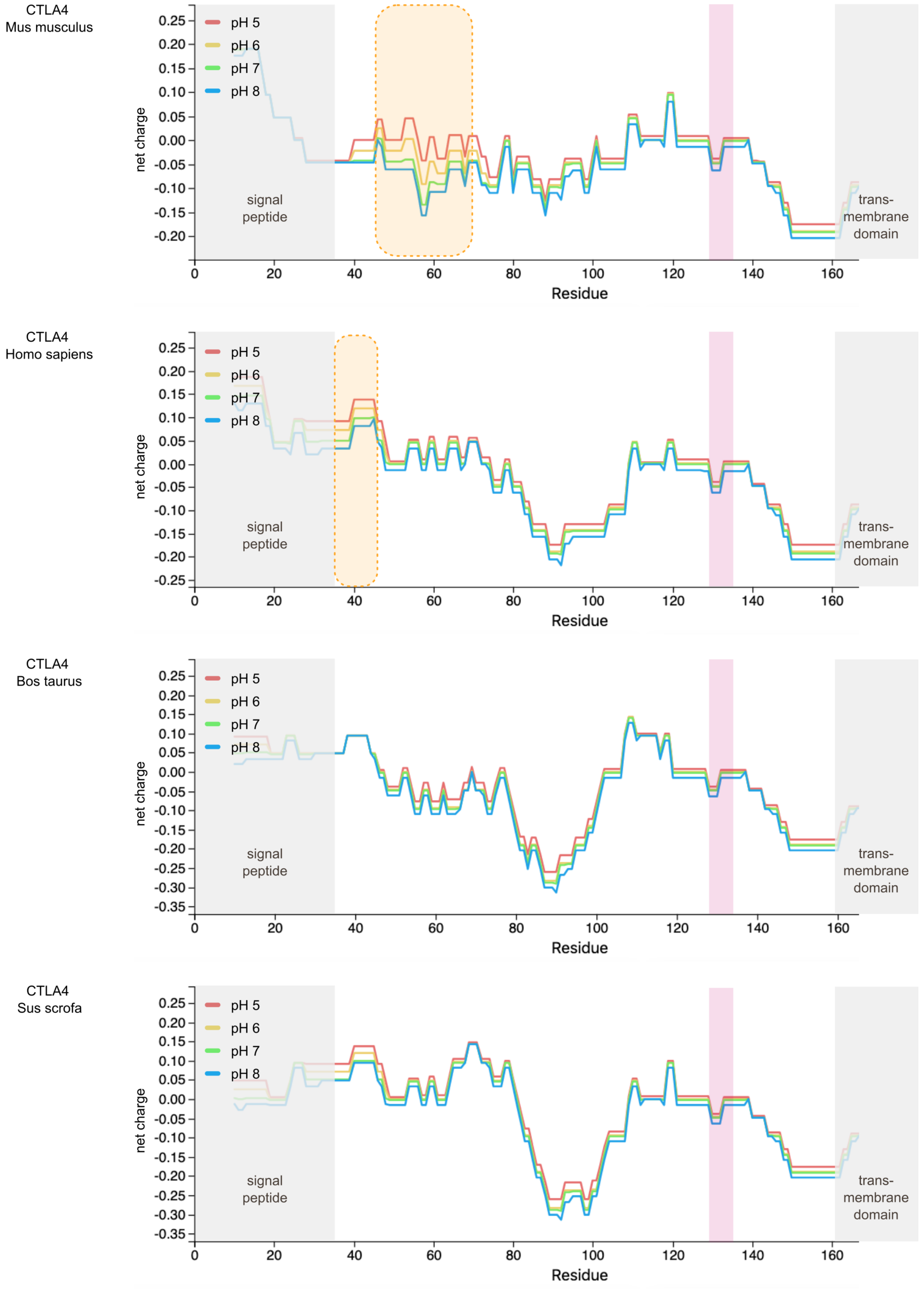

Supplementary Figure 6: Related to Figure 6
The CTLA4 extracellular domain lacks conserved acid-sensitive regions. Charge profiles of the CTLA4 extracellular domain of different species from pH 5 (red) to pH 8 (blue) in steps of pH 1. Sequence regions that exhibit the most pronounced change in charge are marked by an orange rectangle. The MYPPPY-ligand binding motif is tinted pink.

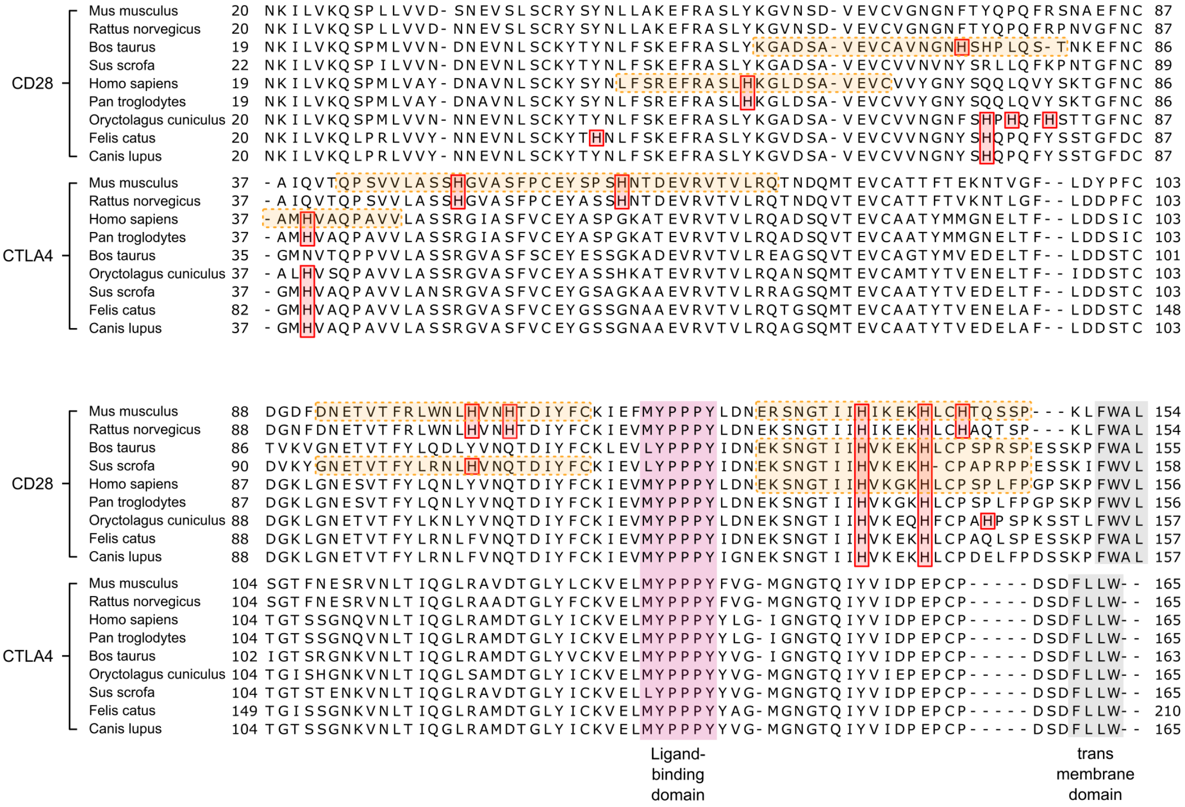

Supplementary Figure 7: Related to Figure 6

The extracellular domain of CD28 contains highly conserved histidines.

Amino acid sequence alignment of CD28 and CTLA4 extracellular domains from different species. Acid sensitive regions identified in Figures S4 and S5 are marked by orange rectangles. Histidines are marked in red. The MYPPPY-ligand binding motif is tinted pink.

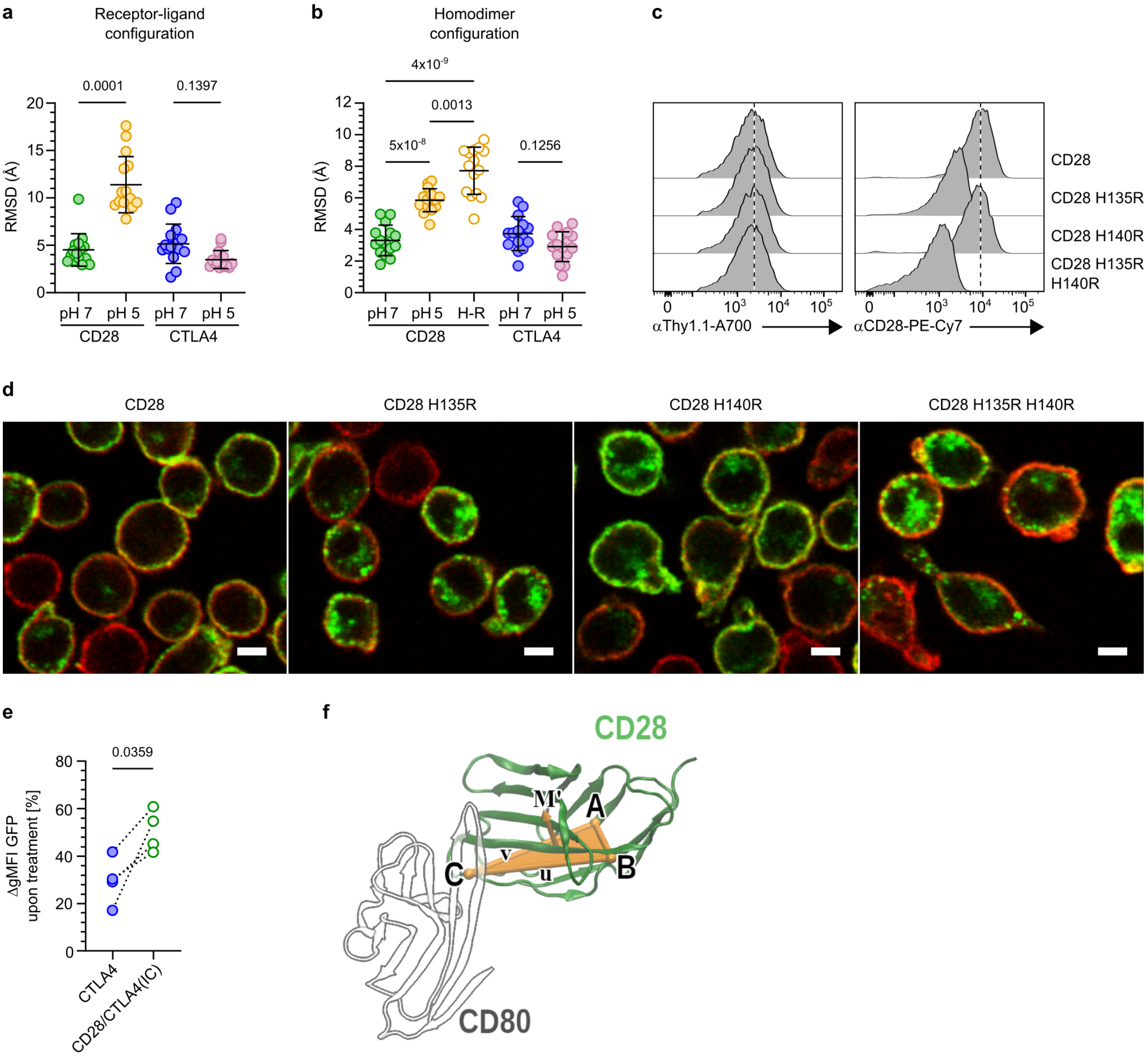

Supplementary Figure 8: Related to Figure 6

Acidification affects CD28 conformation at the ligand-binding and homodimer regions.

(a, b) Root Mean square deviation (RMSD) analysis of molecular dynamic simulations of monomeric CD28 and CTLA4 interacting with CD80 at pH 7 vs. 5 (a) and of the CD28 and CTLA4 homodimer configuration at pH 7 vs. 5 and of a CD28 variant in which both conserved histidines are mutated to arginines (H-R) (b). Data points depict snapshots taken every 2 ns for the last 30 ns of the simulation. (c) Analysis of CHO cells transduced with different CD28-IRES-Thy1.1 constructs. Note that despite comparable Thy1.1 expression, cell surface expression levels of CD28 variants are reduced. (d) Confocal images of $CD28^{-/-}$ T cells transduced to express CD28 variants fused to GFP at their C-terminus. Cell surfaces were stained with WGA-lectin (depicted in red). Overlap of red and green signals is depicted in yellow. Note that for variants containing the H135R mutation a larger fraction of the GFP-signal is located intracellularly. Scale bar 5 µm. (e) Inhibition of acidification stabilizes endosomal conformation of CD28. CTLA4-TagGFP or CD28/CTLA4(IC)-TagGFP expressing 58αβ A2 T cells were cultured with or without Bafilomycin. Plot depicts fold increase of TagGFP upon Bafilomycin treatment.(f) Illustration of parameters and plane used to calculate normal vector of CD28 interacting with CD80.

Statistics: S8a: representative data from 1 of 3 independent MD simulations. Kruskal -Wallis test with Dunn's correction. (n=15 data points per group). S8b: representative data from 1 of 3 independent MD simulations. Brown-Forsythe/Welch's ANOVA with Dunnett's T3 correction. p-values: CD28 pH7 vs. pH5: 5x 10-8, CD28 pH7 vs. H-R: 4x 10-9, CD28 pH5 vs. H-R: 0.0013, CTLA4 pH7 vs. pH5: 0.1256. (n=15 data points per group). S8c: data from 1 experiment. (n=1 biological independent sample per group). S8d: Representative data from 1 of 2 independent experiments. (n=1 biological independent sample per group). S8e: Pooled data from 4 independent experiments. Two-sided paired t test. (n=4 biologically independent samples per group). Source data are provided as a Source Data file.

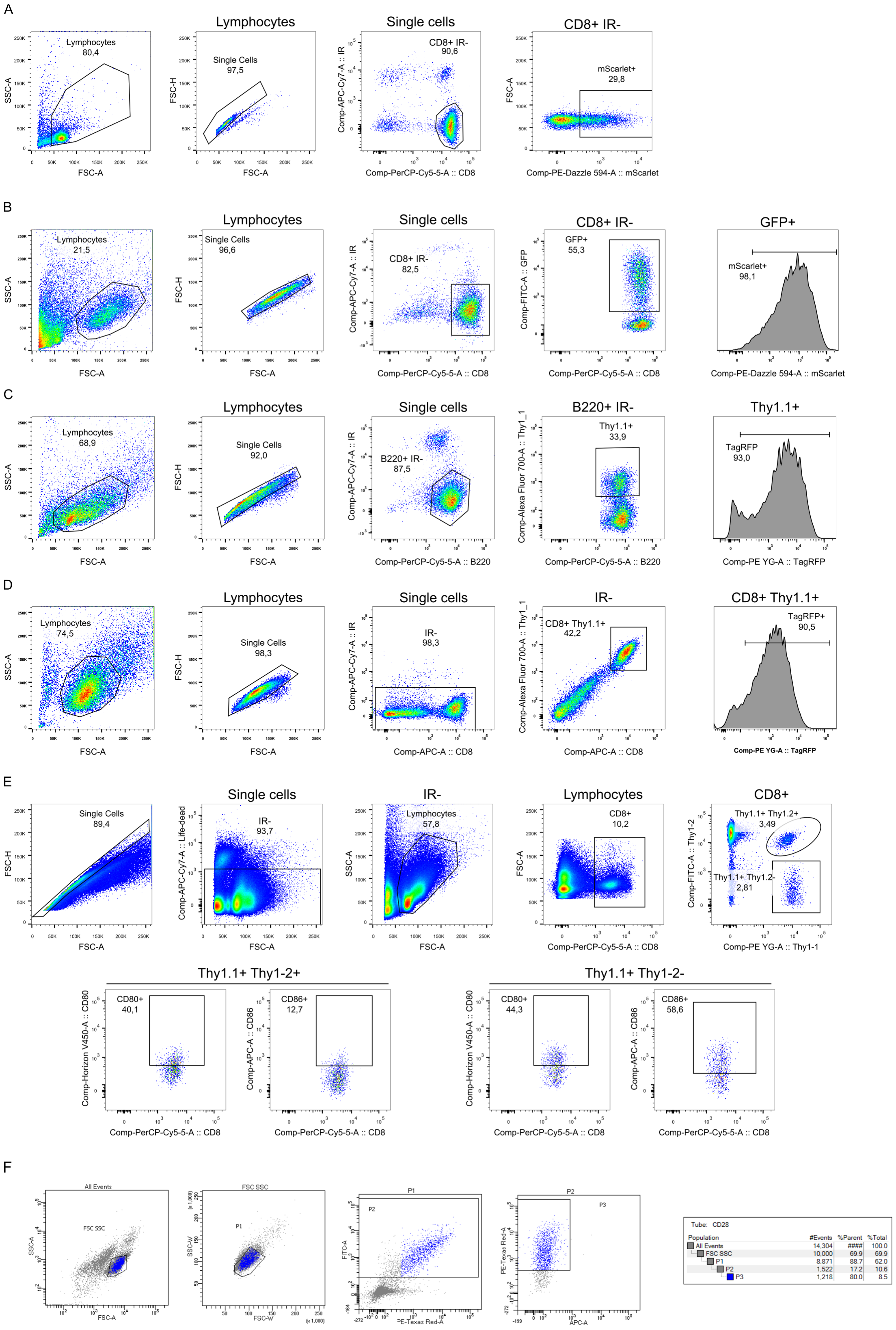

Supplementary Figure 9: Gating strategies

(a) Gating of CD8+ T cells (used for data presented in Figure 1A, D-F; 2A-D, F, G, J). (b) Gating of CD8+ T cells (used for data presented in Figures 2E, F; 4D). (c) Gating of transduced B220+ B cell (used for data presented in Figure S1A). (d) Gating of transduced 58αβ A2 T cell line (used for data presented in Figures 3C, D F; 4B, E, F; 5B-E; 6H-K; S1 B, C; S2 B-D, F, G; S3B; S7C). (e) Gating of T cells upon *in vivo* LCMV infection (used for data presented in Figure 1B). (f) Gate used for FACS-sorting of cells having trogocytosed CD80 (used for cells presented in Figure 2J)
