# Supplementary material for: Differential trafficking of ligands trogocytosed via CD28 versus CTLA4 promotes collective cellular control of co-stimulation
Source: Nat Commun. 2022 Oct 29;13:6459. doi: 10.1038/s41467-022-34156-1 (PMC9617924; doi:10.1038/s41467-022-34156-1)
Supplement: Supplementary file 3 — Description of Additional Supplementary Files [file 41467_2022_34156_MOESM3_ESM.pdf]

Description of Additional Supplementary Files

Supplementary Movie 1
Confocal imaging of CD80 trogocytosis by clustering T cells.

Supplementary Movie 2
3D-visualization of acidification-induced changes in electrostatic potential (ΔE) of CD28 and CTLA4.
