# Supplementary material for: Differential trafficking of ligands trogocytosed via CD28 versus CTLA4 promotes collective cellular control of co-stimulation
Source: Nat Commun. 2022 Oct 29;13:6459. doi: 10.1038/s41467-022-34156-1 (PMC9617924; doi:10.1038/s41467-022-34156-1)
Supplement: Supplementary file 6 — Reporting Summary [file 41467_2022_34156_MOESM6_ESM.pdf]

nature portfolio

Corresponding author(s): Jan Rohr

Last updated by author(s): Sep 10, 2022

Reporting Summary

Nature Portfolio wishes to improve the reproducibility of the work that we publish. This form provides structure for consistency and transparency in reporting. For further information on Nature Portfolio policies, see our Editorial Policies and the Editorial Policy Checklist.

Statistics

For all statistical analyses, confirm that the following items are present in the figure legend, table legend, main text, or Methods section.

n/a	Confirmed	
☐	☒	The exact sample size (*n*) for each experimental group/condition, given as a discrete number and unit of measurement
☐	☒	A statement on whether measurements were taken from distinct samples or whether the same sample was measured repeatedly
☐	☒	The statistical test(s) used AND whether they are one- or two-sided<br>*Only common tests should be described solely by name; describe more complex techniques in the Methods section.*
☒	☐	A description of all covariates tested
☐	☒	A description of any assumptions or corrections, such as tests of normality and adjustment for multiple comparisons
☐	☒	A full description of the statistical parameters including central tendency (e.g. means) or other basic estimates (e.g. regression coefficient) AND variation (e.g. standard deviation) or associated estimates of uncertainty (e.g. confidence intervals)
☐	☒	For null hypothesis testing, the test statistic (e.g. *F*, *t*, *r*) with confidence intervals, effect sizes, degrees of freedom and *P* value noted<br>*Give P values as exact values whenever suitable.*
☒	☐	For Bayesian analysis, information on the choice of priors and Markov chain Monte Carlo settings
☒	☐	For hierarchical and complex designs, identification of the appropriate level for tests and full reporting of outcomes
☐	☒	Estimates of effect sizes (e.g. Cohen's *d*, Pearson's *r*), indicating how they were calculated

Our web collection on statistics for biologists contains articles on many of the points above.

Software and code

Policy information about availability of computer code

Data collection	Fow cytometry data was collected using FACSDiva v9<br>Microscopes where operated using ZEN 3.2 Blue (Zeiss) or LAS-X software (Leica)
Data analysis	Charge distributions of published protein sequences were analyzed using VOLPES (http://volpes.univie.ac.at). Sequences were aligned by Clustal Omega (https://www.ebi.ac.uk/Tools/msa/clustalo/) and visualized by Jailview v2.11.1.1 (https://www.jalview.org/). Isoelectric points of protein extracellular regions were calculated by protparam (https://www.expasy.org/resources/protparam). Molecular dynamic and umbrella sampling simulations were carried out with GROMACS version 2016.5 using the gromos53a6 forcefield. Electrostatic properties of CD28 or CTLA4 complexed with CD80 crystals or modeled complexes were determined with PDB2PQR v3.1.0 software and then analyzed with the Adaptive Poisson-Boltzmann Solver (APBS, Version 1.4.1). 3D illustrations of protein structures were generated by PyMOL Version 2.0<br>Flow cytometry data analysis: FlowJo v10<br>Data analysis: GraphPad Prism v8 & v9<br>Confocal microscopy data analysis: Imaris (v8.3.1 and v9.5.0), Volocity software suite v6.1<br>Figure layout: Inkscape v1.0.1

For manuscripts utilizing custom algorithms or software that are central to the research but not yet described in published literature, software must be made available to editors and reviewers. We strongly encourage code deposition in a community repository (e.g. GitHub). See the Nature Portfolio guidelines for submitting code & software for further information.

Data

Policy information about availability of data

All manuscripts must include a data availability statement. This statement should provide the following information, where applicable:

- Accession codes, unique identifiers, or web links for publicly available datasets
- A description of any restrictions on data availability
- For clinical datasets or third party data, please ensure that the statement adheres to our policy

All data generated or analysed during this study are included in this published article (and its supplementary information files).
Crystal structures of CTLA4 and CD28 extracellular domains were obtained from PDB (PDB 1I85 and PDB 1YJD, respectively). Amino acid sequences of CD28 and CTLA4 were obtained from uniprot (P16410 and P10747, respectively)

Field-specific reporting

Please select the one below that is the best fit for your research. If you are not sure, read the appropriate sections before making your selection.

☒ Life sciences ☐ Behavioural & social sciences ☐ Ecological, evolutionary & environmental sciences

For a reference copy of the document with all sections, see nature.com/documents/nr-reporting-summary-flat.pdf

Life sciences study design

All studies must disclose on these points even when the disclosure is negative.

Sample size	No formal sample size calculation was performed prior to data collection. Sample sizes were chosen based on previous experiences and pilot experiments to yield sample sizes amenable to statistical testing. Our experiments examined the distributions of cellular parameters through single-cell analyses. Sample sizes used provided adequate information about these parameter distributions as demonstrated through reproducibility in replicate experiments.
Data exclusions	In flow-cytometry experiments dead cells were identified by the use of a life/dead-discrimination dye and excluded from analysis.
Replication	Experimental results were reproduced through several independent experiments, the number of which are indicated in the figure legends.
Randomization	For the in vivo experiments recipient mice of adoptively transferred cells were randomly assigned to groups analyzed at different days post transfer. For all in vitro experiments cells were taken from a common homogenous pool and randomly assigned to different treatment groups.
Blinding	No blinding was performed as cells derived from different mouse strains were grouped per genotype. For all other analyses, no blinding was performed as readouts are quantitative and not subjective.

Reporting for specific materials, systems and methods

We require information from authors about some types of materials, experimental systems and methods used in many studies. Here, indicate whether each material, system or method listed is relevant to your study. If you are not sure if a list item applies to your research, read the appropriate section before selecting a response.

Materials & experimental systems

n/a	Involved in the study	
☐	☒	Antibodies
☐	☒	Eukaryotic cell lines
☒	☐	Palaeontology and archaeology
☐	☒	Animals and other organisms
☒	☐	Human research participants
☒	☐	Clinical data
☒	☐	Dual use research of concern

Methods

n/a	Involved in the study	
☒	☐	ChIP-seq
☐	☒	Flow cytometry
☒	☐	MRI-based neuroimaging

Antibodies

Antibodies used	Anti-Mouse CD8a PerCP-eFluor 710 (clone 53-6.7, Thermo Fisher Scientific Cat#46-0081-80, RRID: AB_1834434, 1:333)<br>Anti-Mouse CD8a FITC (clone 53-6.7, Thermo Fisher Scientific Cat#11-0081-85, RRID: AB_464916, 1:400)<br>Anti-Mouse CD8a APC (clone: 53-6.7, Thermo Fisher Scientific Cat#17-0081-82, RRID: AB_10113980, 1:250)<br>Anti-Mouse CD25 BV785 (clone: PC61, BioLegend Cat#102051, RRID: AB_2564131, 1:200)<br>Anti-Mouse CD28 PE-Cy7 (clone: E18, BioLegend Cat#122014, RRID:AB_604079, 1:100)

PE-Cy7 Mouse IgG2b Isotype Control (clone: MPC-11, BioLegend Cat#400325, 1:100)
Anti-Mouse/Human CD44 BV785 (clone: IM7, BioLegend Cat#103059, RRID:AB_2571953, 1:1000)
Anti-Mouse/human CD45R/B220 PerCP/Cyanine5.5 (clone RA3-6B2, BioLegend Cat#103236, RRID:AB_893354, 1:100)
Anti-Mouse CD80 PE/Cy7 (clone 16-10A1, BioLegend Cat#104734, RRID: AB_2563113, 1:200)
Armenian Hamster IgG Isotype Control PE-Cy7 (clone: HTK888, BioLegend Cat#400921, 1:200)
Anti-Mouse CD80 Brilliant Violet 421™ (clone 16-10A1, BioLegend Cat#104726, RRID: AB_2561445, 1:200)
Armenian Hamster IgG Isotype Control Brilliant Violet 421™ (clone: HTK888, BioLegend Cat#400935, 1:200)
Anti-Mouse CD86 APC (clone GL-1, BioLegend Cat#105012, RRID: AB_493342, 1:100)
Rat IgG2a Isotype control APC (clone: RTK2758, BioLegend Cat#400511, 1:100)
Anti-Mouse CD90.1 (Thy1.1) PE (clone: OX-7, BioLegend Cat#202524, RRID:AB_1595524, 1:2000)
Anti-rat CD90/mouse CD90.1 (Thy-1.1) Alexa Fluor 700 (clone: OX-7, BioLegend Cat#202528, AB_1626241, 1:1000)
Anti-Mouse CD90.2(Thy-1.2, FITC (clone: 30-H12) Thermo Fisher Scientific Cat#11090385, RRID:AB_2735034, 1:200)
Anti-Mouse CD90.2 PE (clone: 30-H12, BD Biosciences Cat#553014, AB_394552 1:1000)
Anti-Mouse CD106 APC (clone: 429, BioLegend Cat#105717, RRID:AB_1877142, 1:100)
Anti-Mouse CD152 (CTLA-4) PE (clone UC10-4B9, BioLegend Cat#106306, RRID: AB_313255, 1:200)
Armenian Hamster IgG Isotype Control PE (clone: HTK888, BioLegend Cat#400907, 1:200)
Anti-Mouse TCR Va2 PerCP/Cyanine5.5 (clone: B20.1, BioLegend Cat#127814, RRID:AB_1186116, 1:100)
Anti-Human IgG Alexa Fluor 647 (Fcy fragment specific, Jackson ImmunoResearch Cat#109-605-098, RRID:AB_2337889, 1:400)
Anti-Human IgG Fc APC (clone: M1310G05, BioLegend Cat#410712, RRID:AB_2565790, 1:20)
Anti-LBPA antibody (6C4, Sigma-Aldrich Cat#MABT837, 1:100)
Donkey anti-Mouse IgG (H+L) Alexa Fluor Plus 405 (Thermo Fischer Scientific Cat#A48257, RRID:AB_2884884, 1:400)
Anti-Mouse CD28 LEAF™ Purified (clone 37.51, BioLegend Cat#102112, RRID: AB_312877)
CD28 (clone E18 (51) (produced in house at Institute of Virology and Immunobiology, University of Würzburg, Germany) and CTLA4 (clone UC10-4F10, BioXcell, Cat#BE0032, RRID: AB_1107598) antibody Fab-fragments were generated as described in the Methods section of the manuscript.

Validation

Unless stated otherwise antibodies were validated by manufactures. Antibodies used for flow cytometry were validated using positive and negative staining which is provided in appropriate data sheets available at the following links: CD8a PerCP-eFluor 710 (clone 53-6.7): https://www.thermofisher.com/antibody/product/CD8a-Antibody-clone-53-6-7-Monoclonal/46-0081-80; CD8a FITC (clone 53-6.7): https://www.thermofisher.com/antibody/product/CD8a-Antibody-clone-53-6-7-Monoclonal/11-0081-85;CD8a APC (clone: 53-6.7): https://www.thermofisher.com/antibody/product/CD8a-Antibody-clone-53-6-7-Monoclonal/17-0081-82; CD25 BV785 (clone: PC61): https://www.biolegend.com/en-us/products/brilliant-violet-785-anti-mouse-cd25-antibody-10293; CD28 PE-Cy7 (clone: E18): https://www.biolegend.com/en-us/products/pe-cyanine7-anti-mouse-cd28-antibody-3780; PE-Cy7 Mouse IgG2b Isotype Control (clone: MPC-11): https://www.biolegend.com/en-us/products/pe-cyanine7-mouse-igg2b-kappa-isotype-ctrl-1928; CD44 BV785 (clone: IM7): https://www.biolegend.com/en-us/products/brilliant-violet-785-anti-mouse-human-cd44-antibody-7959; CD45R/B220 PerCP/Cyanine5.5 (clone RA3-6B2): https://www.biolegend.com/en-us/products/percp-cyanine5-5-anti-mouse-human-cd45r-b220-antibody-4267; CD80 PE/Cy7 (clone 16-10A1): https://www.biolegend.com/en-us/products/pe-cyanine7-anti-mouse-cd80-antibody-9320; Armenian Hamster IgG Isotype Control PE-Cy7 (clone: HTK888): https://www.biolegend.com/en-us/products/pe-cyanine7-mouse-igg2b-kappa-isotype-ctrl-1928; CD80 Brilliant Violet 421™ (clone 16-10A1): https://www.biolegend.com/en-us/products/brilliant-violet-421-anti-mouse-cd80-antibody-7357; Armenian Hamster IgG Isotype Control Brilliant Violet 421™ (clone: HTK888): https://www.biolegend.com/en-us/products/brilliant-violet-421-armenian-hamster-igg-isotype-ctrl-7196; CD86 APC (clone GL-1): https://www.biolegend.com/en-us/products/apc-anti-mouse-cd86-antibody-2896; Rat IgG2a Isotype control APC (clone: RTK2758): https://www.biolegend.com/en-us/products/apc-rat-igg2a-kappa-isotype-ctrl-1838; CD90.1 (Thy1.1) PE (clone: OX-7): https://www.biolegend.com/en-us/products/pe-anti-rat-cd90-mouse-cd90-1-thy-1-1-antibody-5620; CD90.1 (Thy-1.1) Alexa Fluor 700 (clone: OX-7): https://www.biolegend.com/en-us/products/alexa-fluor-700-anti-rat-cd90-mouse-cd90-1-thy-1-1-antibody-5883; CD90.2(Thy-1.2) FITC (clone: 30-H12): https://www.thermofisher.com/antibody/product/CD90-2-Thy-1-2-Antibody-clone-30-H12-Monoclonal/11-0903-85; CD90.2 PE (clone: 30-H12): https://www.bdbiosciences.com/en-us/products/reagents/flow-cytometry-reagents/research-reagents/single-color-antibodies-ruo/pe-rat-anti-mouse-cd90-2.553014; CD106 APC (clone: 429): https://www.biolegend.com/en-us/products/apc-anti-mouse-cd106-antibody-6079; CD152 (CTLA-4) PE (clone UC10-4B9): https://www.biolegend.com/en-us/products/pe-anti-mouse-cd152-antibody-516; Armenian Hamster IgG Isotype Control PE (clone: HTK888): https://www.biolegend.com/en-us/products/pe-armenian-hamster-igg-isotype-ctrl-1778; TCR Va2 PerCP/Cyanine5.5 (clone: B20.1): https://www.biolegend.com/en-us/products/percp-cyanine5-5-anti-mouse-tcr-valpha2-antibody-4872; Anti-Human IgG Alexa Fluor 647 (Fcy fragment specific): https://www.jacksonimmuno.com/catalog/products/109-605-098; Anti-Human IgG Fc APC (clone: M1310G05): https://www.biolegend.com/en-us/products/apc-anti-human-igg-fc-11935; Validation of antibodies used for immunofluorescence are available under the following links: Anti-LBPA antibody (clone: 6C4): https://www.sigmaaldrich.com/CH/en/product/mm/mabt837; Donkey anti-Mouse IgG (H+L) Alexa Fluor Plus 405: https://www.thermofisher.com/antibody/product/Donkey-anti-Mouse-IgG-H-L-Highly-Cross-Adsorbed-Secondary-Antibody-Polyclonal/A48257.
Anti-Cd28 antibody clone E18 has been validated in the following publication: Dennehy KM, Elias F, Zeder-Lutz G, Ding X, Altschuh D, Lühder F, et al. Cutting Edge: Monovalency of CD28 Maintains the Antigen Dependence of T Cell Costimulatory Responses. J Immunol. American Association of Immunologists; 2006 May 2;176(10):5725–9.

Eukaryotic cell lines

Policy information about cell lines

Cell line source(s)

Platinum-E retroviral packaging cell line Cell Biolabs Inc. Cat#RV-101, RRID:CVCL_B488
Platinum-A retroviral packaging cell line Cell Biolabs Inc. Cat#RV-102, RRID:CVCL_B489
SV40-immortalized murine embryonic fibroblasts (MEF) were kindly provided from Hartmut Hengel, University of Freiburg, Germany.
BW5147.3 (58αβ) were kindly provided from Wolfgang Schamel, University of Freiburg, Germany.
Chinese Hamster Ovary Cell line (CHO) ATCC Cat#CCL-61, RRID:CVCL_0214

Authentication	Crispr/Cas9-mediated knockout of CD28 expression was authenticated by flow cytometric analysis. None of the other cell lines used were authenticated in our lab.
Mycoplasma contamination	All cell lines were tested negative for mycoplasma contamination.
Commonly misidentified lines (See ICLAC register)	No commonly misidentified cell lines were used.

Animals and other organisms

Policy information about studies involving animals; ARRIVE guidelines recommended for reporting animal research

Laboratory animals	P14 TCR-transgenic mice (TcrLCMV)318Sdz/JDvsJ)<br>Cd80-/-Cd86-/- (B6.129S4-Cd80tm1Shr Cd86tm2Shr/J)<br>mTmG (B6.129(Cg)-Gt(ROSA)26Sortm4(ACTB-tdTomato,-EGFP)Luo/J)<br>C57BL/6<br>Cd28-/- (B6.129S2-Cd28tm1Mak/J)<br>Ctla4-/- OT-I (CTLA4-/- described in PMID: 9256476 were crossed to OT-I mice (C57BL/6-Tg(TcraTcrb)1100Mjb/J))<br>Mice from both sexes were used in this study, but within experiments sex-matched mice were used. Age: 6-22 weeks. Mice were bred and maintained under SPF conditions in accredited animal facilities. Animals were housed in individually ventilated cages with 12h dark/light cycle at a temperature of 20-23°C, and supplied with food and water ad libitum.
Wild animals	The study did not involve wild animals.
Field-collected samples	The study did not involve samples collected from the field.
Ethics oversight	Animal experiments were approved by local governmental authorities (Regierungspräsidium Freiburg) and performed in accordance with EU guidelines.

Note that full information on the approval of the study protocol must also be provided in the manuscript.

Flow Cytometry

Plots

Confirm that:

- [x] The axis labels state the marker and fluorochrome used (e.g. CD4-FITC).
- [x] The axis scales are clearly visible. Include numbers along axes only for bottom left plot of group (a 'group' is an analysis of identical markers).
- [x] All plots are contour plots with outliers or pseudocolor plots.
- [x] A numerical value for number of cells or percentage (with statistics) is provided.

Methodology

Sample preparation	Murine splenic B and CD8+ T cells were magnetically purified (Mouse CD8 T lymphocyte enrichment set – DM, BD Biosciences and MojoSort™ Mouse Pan B Cell Isolation Kit, BioLegend).<br>Blood lymphocytes were enriched by erythrocyte lysis (BD FACS™ Lysing Solution, BD Biosciences). Lungs were perfused with PBS and digested with 300 U/ml collagenase II (Thermo Fisher Scientific) and 10 U/ml DNAse I (Sigma-Aldrich) in medium supplemented with 25mM magnesium chloride (Sigma-Aldrich) for 1h at 37°C. Spleens, lymph nodes and livers were dispersed using 70µm cell strainers (Greiner). Lymphocytes from liver and lung suspensions were isolated by density gradient centrifugation (Lympholyte®-M, Cedarlane).
Instrument	Becton Dickinson FACS Fortessa<br>Becton Dickinson FACS Aria Fusion (cell sorting)<br>Becton Dickinson FACS AriaIII (cell sorting)<br>All flow cytometers are equipped with 405/488/561/640nm lasers
Software	Data was collected using FACSDiva v9 software and analysed using FlowJo v10 (both from BD Biosciences)
Cell population abundance	Post-sort quality control yielded > 97% purity of cells.
Gating strategy	For FACS-analysis:<br>All samples were gated for lymphocytes (FSC-A, SSC-A), single cells (FSC-A, FSC-H), live cells (IR Live/Dead marker negative).<br><br>Trogocytosis by lymphocytes in in vitro co-culture assays:<br>Primary T cells: CD8+, mScarlet+<br>Transduced T cells: CD8+, GFP+ , mScarlet+ or TagRFP+ (depending on fusion protein used)<br>Transduced B cells: B220+, Thy1.1+, mScarlet+ or TagRFP+ (depending on fusion protein used)<br>58αβ T cell line: CD8+, Thy1.1+, mScarlet+ or TagRFP+ (depending on fusion protein used)

Trogocytosis in vivo: single cells (FSC-A, FSC-H), live cells (IR Live/Dead marker negative), lymphocytes (FSC-A, SSC-A), CD8+, transferred cells (Thy1.1+, Thy1.2+/-), CD80+, CD86+

Cell sorting: samples were gated for lymphocytes (FSC-A, SSC-A), single cells (FSC-W, SSC-W), transduced cells (GFP+), mScarlet+, CD106-APC-

☒ Tick this box to confirm that a figure exemplifying the gating strategy is provided in the Supplementary Information.
